# *Mycobacterium bovis* Strain Ravenel Is Attenuated in Cattle

**DOI:** 10.3390/pathogens11111330

**Published:** 2022-11-11

**Authors:** Syeda A. Hadi, Evan P. Brenner, Mitchell V. Palmer, W. Ray Waters, Tyler C. Thacker, Catherine Vilchèze, Michelle H. Larsen, William R. Jacobs, Srinand Sreevatsan

**Affiliations:** 1 Pathobiology and Diagnostic Investigation Department, Michigan State University, East Lansing, MI 48824, USA; 2 National Animal Disease Center, Agricultural Research Service, US Department of Agriculture, Ames, IA 50010, USA; 3 National Veterinary Services Laboratories, US Department of Agriculture, Ames, IA 50010, USA; 4 Department of Microbiology and Immunology, Albert Einstein College of Medicine, Bronx, NY 10475, USA

**Keywords:** tuberculosis, *Mycobacterium bovis*, attenuation, pathogenomics, strain Ravenel, bovine TB, SNPs

## Abstract

*Mycobacterium tuberculosis* variant *bovis* (MBO) has one of the widest known mammalian host ranges, including humans. Despite the characterization of this pathogen in the 1800s and whole genome sequencing of a UK strain (AF2122) nearly two decades ago, the basis of its host specificity and pathogenicity remains poorly understood. Recent experimental calf infection studies show that MBO strain Ravenel (MBO Ravenel) is attenuated in the cattle host compared to other pathogenic strains of MBO. In the present study, experimental infections were performed to define attenuation. Whole genome sequencing was completed to identify regions of differences (RD) and single nucleotide polymorphisms (SNPs) to explain the observed attenuation. Comparative genomic analysis of MBO Ravenel against three pathogenic strains of MBO (strains AF2122-97, 10-7428, and 95-1315) was performed. Experimental infection studies on five calves each, with either MBO Ravenel or 95-1315, revealed no visible lesions in all five animals in the Ravenel group despite robust IFN-γ responses. Out of 486 polymorphisms in the present analysis, 173 were unique to MBO Ravenel among the strains compared. A high-confidence subset of nine unique SNPs were missense mutations in genes with annotated functions impacting two major MBO survival and virulence pathways: (1) Cell wall synthesis & transport [*espH* (A103T), *mmpL8* (V888I), *aftB* (H484Y), *eccC*_5_ (T507M), *rpfB* (E263G)], and (2) Lipid metabolism & respiration [*mycP_1_*(T125I), *pks5* (G455S), *fadD*29 (N231S), *fadE29* (V360G)]. These substitutions likely contribute to the observed attenuation. Results from experimental calf infections and the functional attributions of polymorphic loci on the genome of MBO Ravenel provide new insights into the strain’s genotype-disease phenotype associations.

## 1. Introduction

*Mycobacterium tuberculosis* variant *bovis* (*Mycobacterium bovis* or MBO) causes significant economic hardship for livestock producers. *M. bovis*, a member of the *Mycobacterium tuberculosis* complex, is infectious to humans [1,2] and causes ~150,000 cases of human disease annually [3]. However, the overall virulence of MBO is generally greater than that of *M. tuberculosis* [4,5,6,7], as is reflected in the extensive animal host range. 

Historically, inoculation of rabbits was used to discriminate *M. tuberculosis variant tuberculosis* (MTB) from MBO within clinical samples; rabbits are typically resistant to MTB but highly susceptible to MBO infection. In contrast, guinea pigs and mice are susceptible to both MTB and MBO.

The MBO type strain for many comparative pathogenesis studies is *M. bovis* Ravenel (ATCC strain 35720). This strain was isolated from a tuberculous cow circa 1900 and deposited in the Trudeau Mycobacterial Culture Collection in 1910. At the Trudeau Institute, this strain was maintained in the non-native host rabbit before freezer stocks were made. Here it was used for pathogenesis and virulence studies in mice, rabbits, and guinea pigs. It has been used in diagnostic [8], immunopathogenesis [5,6], chemotherapy [9], and vaccine efficacy studies [10,11]. Even though the current stocks of MBO Ravenel (ATCC strain 35720) are fully virulent in rabbits [12,13], guinea pigs [13], and mice [13,14,15] yet they are attenuated in cattle. 

Experimental infection of calves with MBO strains such as 95-1315 at the National Animal Disease Center (NADC) in the US [16], WAg202 at AgResearch in New Zealand [17], and AF2122/97 at Animal Health and Veterinary Laboratory Agency (AHVLA) in the United Kingdom [18,19] consistently result in progressive disease in cattle, including typical granulomatous gross and microscopic lesions with recovery of bacilli from affected tissues. However, in a bovine TB vaccine efficacy study with cattle by Khare et al., 2007 [11], gross tuberculous lesions were not detected in vaccinated or unvaccinated groups upon necropsy 160 days after intranasal delivery of 10^6^ colony forming units (CFU) of MBO Ravenel challenge, despite the recovery of MBO Ravenel from 10/10 unvaccinated and 4/10 vaccinated animals. However, histopathological changes were seen in 6/10 unvaccinated as well as 4/10 vaccinated animal’s tonsils and livers. Even though all the referenced studies used different infection routes, dose rates, ages, and animal breeds, the fact remains that Ravenel failed to replicate the macroscopic granulomatous lesions seen in infection with other pathogenic strains of MBO. Because of this deficiency, pathogenesis studies no longer employ MBO Ravenel. Given these reports on apparent disease phenotype variations among different strains of MBO, the objectives of the present study were: (1) to compare the virulence of MBO Ravenel to MBO strain 95-1315 in cattle and (2) to compare the genome sequences of MBO Ravenel to three virulent strains of MBO. 

## 2. Methods

### 2.1. Mycobacterium Tuberculosis Variant Bovis Challenge Strains

Two strains of MBO were used for the challenge inoculum: (1) MBO strain 95-1315 [USDA, Animal Plant and Health Inspection Service (APHIS) designation] originally isolated from a white-tailed deer in Michigan [20], USA and (2) MBO strain Ravenel (ATCC 35720) obtained from John Chan at Albert Einstein College of Medicine, Bronx, NY and freezer stocks were kept at NADC. Strains were prepared using standard procedures [21] in Middlebrook 7H9 liquid media (Becton Dickinson, Franklin Lakes, NJ, USA) supplemented with 10% oleic acid-albumin-dextrose complex (OADC) plus 0.05% Tween 80 and 0.5% Glycerol (strain Ravenel only). 

### 2.2. Cattle Studies: Treatment Groups and Aerosol MBO Challenge Procedures

Holstein steers (*n* = 18, ~1 year of age) were obtained from a TB-free herd in Sioux Center, IA, and housed in a biosafety level-3 (BSL-3) facility at the NADC, Ames, IA, according to Institutional Biosafety and Animal Care and Use Committee guidelines. In an initial study, steers (*n* = 3) received 10^5^ CFU MBO Ravenel by aerosol. Briefly, inoculum (~10^5^ CFU) was delivered to restrained calves by nebulization into a mask (Trudell Medical International, London, ON, Canada) covering the nostrils and mouth. The inoculum was inhaled through a one-way valve into the mask and directly into the lungs via the nostrils. The process continued until the inoculum, a 1 mL PBS wash of the inoculum tube, and an additional 2 mL PBS were delivered, taking ~10 min. Strict BSL-3 safety protocols were followed to protect personnel from exposure to MBO.

In a follow-up study, treatment groups consisted of uninfected steers (*n* = 5) and steers receiving either 10^5^ CFU MBO strain 95-1315 (*n* = 5) or 10^5^ CFU MBO strain Ravenel (*n* = 5) by aerosol [16].

All calves were euthanized ~3.5 months after challenge by intravenous administration of sodium pentobarbital. Tissues were examined for gross lesions and collected and processed for microscopic analysis and isolation of MBO. Tissues collected included: palatine tonsil, lung, liver, and mandibular, parotid, medial retropharyngeal, mediastinal, tracheobronchial, hepatic, and mesenteric lymph nodes. Lymph nodes were sectioned at 0.5 cm intervals and examined. Each lung lobe was sectioned at 0.5–1.0 cm intervals and examined separately. Lungs and lymph nodes (mediastinal and tracheobronchial) were evaluated using a semi-quantitative gross pathology scoring system adapted from Vordermeier et al., 2002 [17]. Lung lobes (left cranial, left caudal, right cranial, right caudal, middle, and accessory) were individually scored based upon the following scoring system: 0 = no visible lesions; 1 = no external gross lesions, but lesions seen upon slicing; 2 = <5 gross lesions of <10 mm in diameter; 3 = >5 gross lesions of <10 mm in diameter; 4 = >1 distinct gross lesion of >10 mm in diameter; 5 = gross coalescing lesions. Cumulative mean scores were then calculated for each entire lung. Lymph node pathology was based on the following scoring system: 0 = no necrosis or visible lesions; 1 = small focus (1 to 2 mm in diameter); 2 = several small foci; 3 = extensive necrosis. Gross pathology data are presented as mean (±standard error) disease score for mediastinal lymph node, tracheobronchial lymph node, and lung. 

Tissues collected for microscopic analysis were fixed by immersion in 10% neutral buffered formalin. For microscopic examination, formalin-fixed tissues were processed by standard paraffin-embedment techniques, cut into 5 µm sections, and stained with hematoxylin and eosin (H&E). Adjacent sections from sections containing caseous necrotic granulomas suggestive of tuberculosis were cut and stained by the Ziehl–Neelsen technique for visualization of acid-fast bacteria (AFB). Microscopic tuberculous lesions were staged (I–IV) as described by Wangoo [22]. Data are presented as the total and mean number of granulomas observed in each histologic lesion stage (i.e., I–IV) for lung and mediastinal lymph node sections (Table 1).

For quantitative assessment of mycobacterial burden, left tracheobronchial lymph nodes were removed, examined for gross lesions, weighed, and the entire lymph node (other than a small ~1 g section for histology and qualitative culture) homogenized in phenol red nutrient broth using a blender (Oster, Shelton, CT, USA). Logarithmic dilutions (10^0^–10^−9^) of homogenates in PBS were plated in 100μL aliquots plated on Middlebrook 7H11 selective agar plates (Becton Dickinson) and incubated for 8 weeks at 37 °C [23]. Data are presented as mean (±standard error) CFU per gram of tissue (Table 2). 

### 2.3. IFN-γ Whole Blood Assays

**Figure 1:**
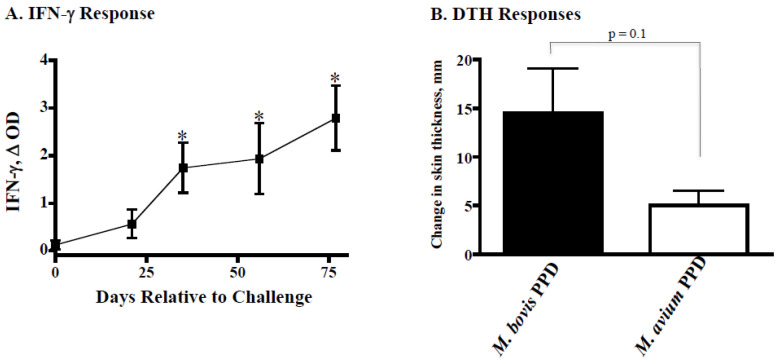
Interferon-γ (panel (**A**)) and delayed type hypersensitivity (DTH, panel (**B**)) responses upon aerosol *M. bovis* Ravenel (*n* = 3) infection. *Panel A*. Whole blood cultures were stimulated with 20 mg/mL *M. bovis* PPD (Prionics AG) or medium alone (no stimulation) for 48 h. Stimulated plasma were harvested, and IFN-γ concentrations were determined by ELISA (Bovigam, Prionics, Ag). Values represent mean (±standard error) responses to antigen minus the response to media alone (D OD) for *M. bovis* Ravenel-infected cattle. Response kinetics to rESAT-6/CFP10 were similar to *M. bovis* PPD responses (data not shown). * Differs (*p* < 0.05) from pre-challenge (Day 0) responses. *Panel B*. Approximately 2 months after challenge, 0.1 mL (100 mg) of *M. bovis* PPD and 0.1 mL (40 mg) of *M. avium* PPD were injected intradermally at separate clipped sites in the mid-cervical region of each calf. Values represent mean (±standard error) change in skin thickness (i.e., 72 h post-injection minus pre-injection).

**Figure 2:**
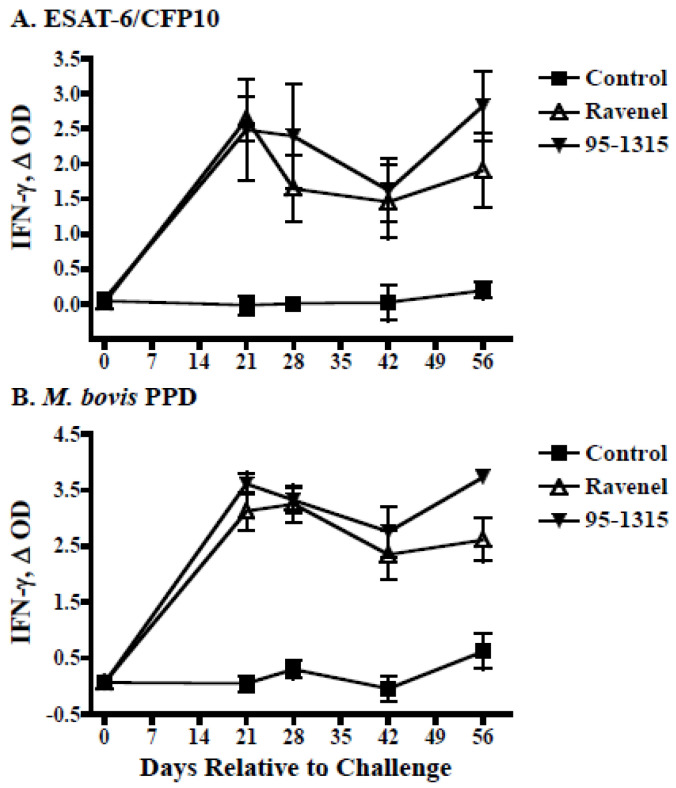
Interferon-γ responses upon *M. bovis* infection of cattle. Whole blood cultures were stimulated with 1 mg/mL rESAT-6:CFP-10 (panel (**A**)), 20 mg/mL *M. bovis* PPD (panel (**B**)), or medium alone (no stimulation) at 39 °C/5% CO_2_ for 20 h. Stimulated plasma were harvested, and IFN-γ concentrations were determined by ELISA (Bovigam, Prionics, Ag). Values represent mean (±standard error) responses to antigen minus the response to media alone (D OD) for non-infected cattle (controls, closed squares) or cattle infected with *M. bovis* strain Ravenel (open triangles) or strain 95-1315 (closed inverted triangles). All responses elicited after challenge with *M. bovis* (10^5^ CFU MBO Ravenel by aerosol either strain) exceeded (*p* < 0.05) respective responses in non-infected (control) cattle. Responses elicited by *M. bovis* Ravenel infection did not differ (*p* > 0.05) from responses elicited by *M. bovis* 95-1315.

Duplicate 250 µL heparinized whole blood aliquots were distributed in 96-well plates with MBO purified-protein derivates (PPD) (10 µg/mL, Prionics Ag, Schlieren, Switzerland), rESAT-6/CFP10 (1 µg/mL), or no antigen (nil) and incubated at 39 °C/5% CO_2_ for 20 h. IFN-γ concentrations in stimulated plasma were determined using a commercial ELISA-based kit (Bovigam^TM^, Prionics Ag). Absorbance values of standards (recombinant bovine IFN-γ; Endogen, Rockford, IL, USA) and test samples were read at 450 nm using an ELISA plate reader (Molecular Devices, Menlo Park, CA, USA). Duplicate samples for individual treatments were analyzed, and data presented as optical densities at 450 nm of the response to MBO PPD minus the response to no-antigen (mean ± SEM) (Figure 1 and Figure 2).

### 2.4. Delayed Type Hypersensitivity (DTH) Responses (Skin Test Procedures)

**Figure 3:**
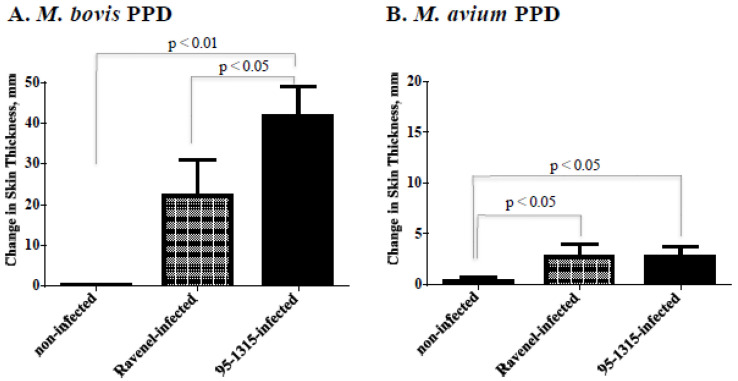
Delayed type hypersensitivity responses upon aerosol *M. bovis* infection. Approximately 3.25 months after challenge, 0.1 mL (100 mg) of *M. bovis* PPD and 0.1 mL (40 mg) of *M. avium* PPD were injected intradermally at separate clipped sites in the mid-cervical region of each calf according to USDA, APHIS uniform methods (APHIS 91-45-011). Values represent mean (±standard error) change in skin thickness (i.e., 72 h post injection minus pre-injection). Differences between treatment groups are indicated on each graph.

Fifteen days prior to necropsy, calves received 0.1 mL (100 µg) of MBO PPD and 0.1 mL (40 µg) of *Mycobacterium avium* PPD injected intradermally at separate clipped sites in the mid-cervical region according to guidelines described in USDA, Animal and Plant Health Inspection Service (APHIS), Veterinary Services (VS) circular 91-45-01 (APHIS, 2005) for the comparative cervical test. Skin thickness was measured with calipers prior to PPD administration and 72 h after injection (Figure 3). A scattergram for the interpretation of CCT results provided by USDA, APHIS, and VS was used to categorize animals as negative, suspect, or reactors. Balanced PPDs were obtained from the Brucella and Mycobacterial Reagents section of the National Veterinary Services Laboratory, Ames, IA, USA.

### 2.5. Whole Genome Sequencing of MBO Ravenel

**Figure 4:**
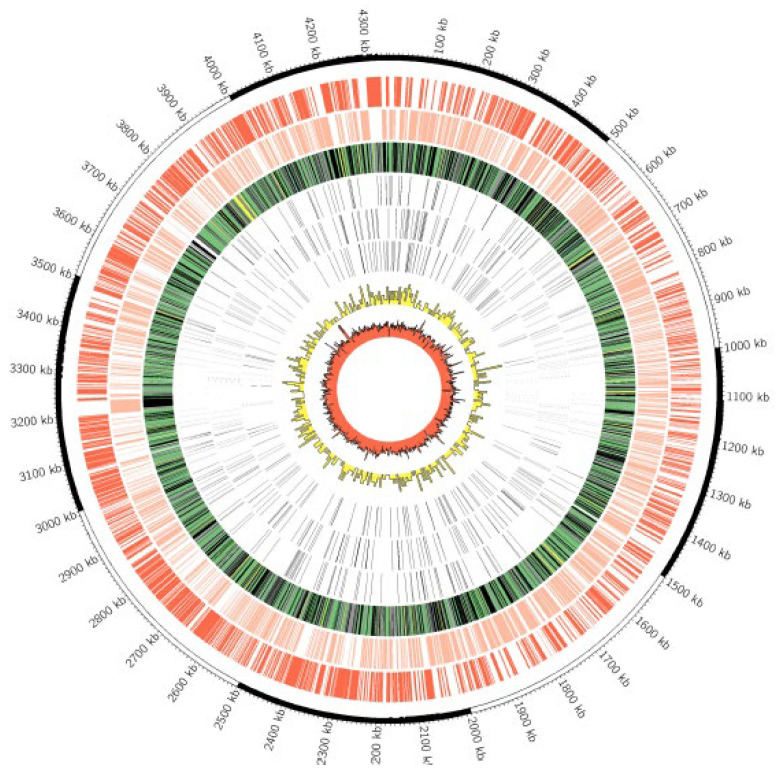
**Genome-wide comparisons of Ravenel and 95-1315.** The outermost circle is the scale in kilobase pairs. The 1st two rings depict coding regions in the positive and negative strands, respectively. The 3rd ring depicts the predicted subcellular localization of each protein, with blue = cell wall, green = cytoplasmic, black = cytoplasmic membrane, yellow = extracellular, and grey = unknown. Rings 4–6 depict SNPs that are shared by both strains Ravenel and 95-1315 (Ring 4), unique to strain 95-1315 (Ring 5), and unique to strain Ravenel (Ring 6). Ring 7 in yellow depicts SNP density across the *M. bovis* genome in 10-kb increments. Ring 8 in red depicts G+C content in a 3-kb sliding window.

**Figure 5:**
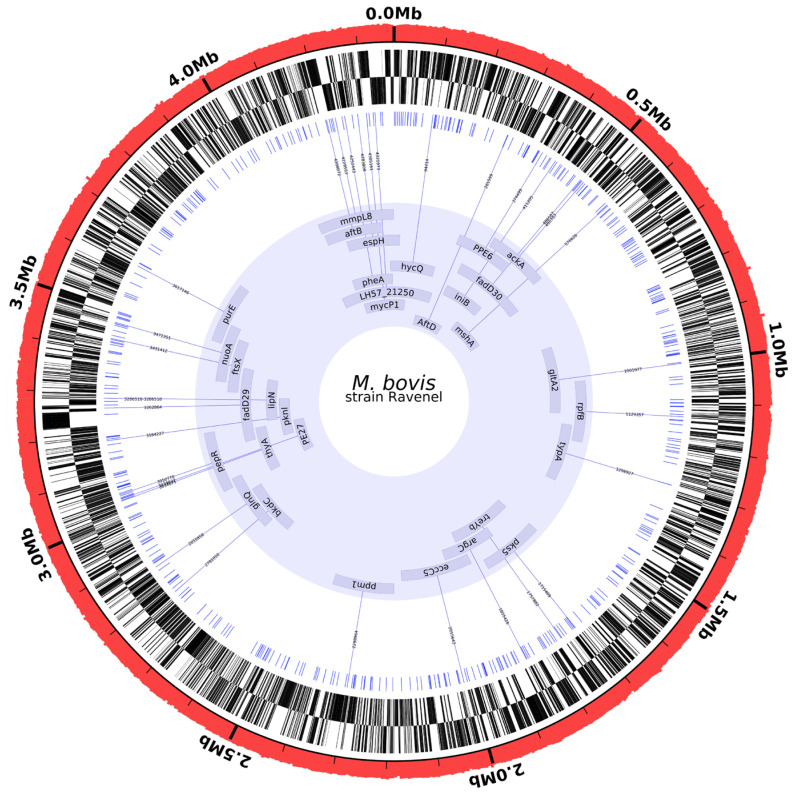
**Position of 32 high-confidence missense polymorphisms in the Ravenel assembly.** The Ravenel genome assembly (GCA_018305025.1) was downloaded, and contigs longer than 1000 bp were visualized using Circleator. Working inwards, the rings represent: red, genome %GC; black, genes-fwd; black, genes-rev; blue, total unique polymorphisms (*n* = 486); central blue ring with labeled loci, 32 high-confidence missense polymorphisms in genes with annotated function. Of these polymorphisms, 31 are SNPs, and 1 is a multi-nucleotide polymorphism (lipN). The manuscript discusses nine SNPs in this innermost subset in greater detail.

MBO Ravenel was obtained from freezer stocks stored at NADC (APHIS, USDA) and cultured on Middlebrook 7H10 slants (Hardy Diagnostics, Santa Maria, CA, USA) for 14 days. Colonies were harvested for DNA extraction. Paired-end (2 × 150 bp) library preparation using NEBNext DNA library preparation kit (New England BioLabs, Ipswich, MS, USA) was followed by NovaSeq Illumina genome sequencing (Novogene) (https://www.ncbi.nlm.nih.gov/sra/SRX10318108; accessed on 7 April 2021). All reads were quality-checked, and adapters were trimmed by Novogene’s in-house custom software (v1.0). Sequences were then checked for contamination using Kraken 2 with default parameters and the author-provided “Standard” database [24]. All reads were used to assemble the genome de novo, irrespective of Kraken’s taxonomy assignment. ABySS v2.1.5 (k-value = 96) was used to assemble the genome [25]. RagTag v1.1.0 was used to perform MBO AF2122/97 (GenBank accession number LT708304) reference-based assembly correction followed by scaffolding [24]. QUAST v5.0.2 was used to analyze scaffolds [25] and generate Circos plots [26] and Icarus views (Figure 4 and Figure 5—Circos-Images). Uninformative contigs (<200 bp) were removed before submission to the Prokaryotic Genome Annotation Pipeline (PGAP) v5.1 [27]. The draft genome was submitted to both PATRIC [28] and NCBI. 

### 2.6. Genome Comparisons

#### 2.6.1. Identification of Single Nucleotide Polymorphisms (SNPs) in MBO Ravenel 

MBO Ravenel’s genomic comparisons were performed against the reference strain MBO AF2122/97, MBO 95-1315 (isolated from deer, PATRIC ID 1765.15), and MBO 10-7428 (isolated from cattle). MBO 10-7428 was submitted to Novogene for resequencing as described above, assembled as a draft genome (PATRIC ID 1765.618; GenBank: JAGEUC000000000.1), and used for comparative genomics and SNP extraction. Snippy [29] was used with default parameters (variant site coverage ≥ 10 reads, VCF call quality = 100, read mapping quality ≥ 60, base quality ≥ 13) to call polymorphisms and indels from paired-end reads from MBO Ravenel (SRR13938830), MBO 10-7428 (SRR13938829) and MBO 95-1315 using MBO AF2122/97 as the reference. The snippy-core script extracted polymorphisms at all positions with sufficient coverage for base calling from all four genomes. Those not unique to MBO Ravenel were removed from the analysis. Indels were placed into a separate data table. The SNPs that remained at this stage were unique to MBO Ravenel. Among these, only the missense mutations in genes with a putative function assigned were cross-checked against the annotated genome on PATRIC. 

#### 2.6.2. Region of Difference (RD) and Gene Presence/Absence Analysis

RD-Analyzer [30] was used to identify in silico regions of difference for MBO Ravenel when compared against MBO AF2122/97 using raw reads of the available genome. To check for deletions outside of characterized RDs, contigs for MBO Ravenel and the genomic .fasta sequence for MBO AF2122/97 were annotated using prokka [https://github.com/tseemann/prokka; accessed on 10 January 2021], and output .gff files analyzed by GenAPI [https://github.com/MigleSur/GenAPI; accessed on 11 November 2020] with default settings. Further validation of gene absence in silico was performed using NCBI’s BLAST [https://pubmed.ncbi.nlm.nih.gov/24642063/; accessed on 12 November 2020].

## 3. Results

### 3.1. Experimental Infection of Cattle

In an initial study with cattle (*n* = 3), aerosol MBO Ravenel (~10^5^ CFU) elicited immune responses (i.e., DTH and IFN-γ) to MBO antigens (Figure 1); yet, 2.5 months after challenge, tuberculous lesions were not detectable in two animals, and a single small granuloma was detected in the lung of the third animal. 

To confirm this finding, an immunopathogenesis study was performed to directly compare the virulence of MBO strain Ravenel (*n* = 5) to that of MBO strain 95-1315 (*n* = 5), a strain of consistent virulence in cattle [15,16]. Significant IFN-γ responses to rESAT-6/CFP10 (E:C) or MBO PPD were elicited by MBO infection (Figure 2), regardless of strain. IFN-γ responses did not differ (*p* > 0.05) between the two challenge groups. Significant DTH responses to PPDs were also elicited by MBO infection (Figure 3). Responses to MBO PPD by MBO 95-1315 infected calves exceeded (*p* < 0.05) respective responses by MBO Ravenel infected calves. 

Despite robust cell-mediated immune (CMI) responses, gross tuberculous lesions were not detectable in 4/5 MBO Ravenel-infected cattle (Table 1). As with the initial study, one MBO Ravenel-infected steer had a single small granuloma in the left caudal lung lobe. In contrast, all 5 MBO 95-1315-infected cattle had granulomatous lesions in the lungs and lung-associated lymph nodes. Microscopic examination revealed no additional granulomas in the tissues collected.

In contrast to gross and microscopic pathology findings, MBO Ravenel was isolated from tissues of 4/5 cattle (Table 2). Tracheobronchial lymph nodes were the most common site for detectable MBO Ravenel colonization, despite no observable gross lesions in that anatomic site. As expected from prior studies, MBO 95-1315 was isolated from lungs and lung-associated lymph nodes from all five calves receiving this strain. Mean colonization of tracheobronchial lymph nodes in MBO 95-1315-infected calves exceeded that of MBO Ravenel-infected calves. 

### 3.2. Whole Genome Sequencing

The MBO Ravenel genome has been assembled and deposited in publicly accessible databases (GenBank: JAGEUB000000000.1; PATRIC: 1765.617). The sequencing yielded 9,074,522 spots (2 × 150 bp/spot). Draft assembly of the genome after removal of uninformative contigs (<200 bp) yielded 18 final contigs. The total length of the genome was 4,377,551 bp, with a GC percentage of 65.6%. The length of the longest contig (N50) was 4,371,545 bp with a coverage of 625.8×. The NCBI-based Prokaryotic Genome Annotation Pipeline (PGAP) identified 4058 coding sequences (CDSs), 3 rRNAs, 45 tRNAs, 3 noncoding RNAs, and 192 pseudogenes. 

### 3.3. Genome Comparisons

#### SNPs from MBO Ravenel versus Virulent MBO Strains 

Comparisons between strains AF2122/97, 10-7428, Ravenel, and 95-1315 revealed no large-scale deletions in the Ravenel genome compared to the AF2122/97 genome. A total of 974 single nucleotide polymorphisms (SNPs) were extracted from the three strains against the AF2122/97 reference. Out of 974 SNPs in the set, 173 were present in and unique to MBO Ravenel. Of these, 95 were missense, and 54/95 missense mutations were in genes with a putative function assigned. Regions of the genome assembly flagged by QUAST as having potential for any degree of mis-assembly were excluded, leaving a subset of 32 highest-confidence SNPs *(*Figure 4 and Figure 5, and Ravenel Supplementary Table S1). Nine highest-confidence SNPs were within “specialty genes” (a PATRIC-defined category of essential genes, virulence factors, AMR-associated genes, and others) in the functionally annotated genome available on PATRIC (1765.617) (Table 3). These nine SNPs were cross-checked in Mycobrowser for their functional categorization [31]. Two functional categories were identified: (i) cell wall and cell processes (*espH*, *mmpL*8, *aftB*, *eccC_5_*, *rpf*B), and (ii) respiration or lipid metabolism (*mycP_1_*, *pks5*, *fadD29*, *fadE29*).

Region of difference (RD) analysis of MBO Ravenel revealed that RD9, RD4, RD7, RD8, RD10, RD11, and RD12 were absent (classic MBO type). GenAPI reported seven total genes absent in MBO Ravenel and present in MBO AF2122/97, five of which fall into PE/PPE family proteins and may be false negatives due to the inherent difficulties in Illumina sequencing these regions, one prokka false positive for a short hypothetical protein not annotated by PGAP in the reference, and one showing a loss of the LuxR-family transcriptional regulator Mb2515c. This latter deletion was checked with 14,000 nt of sequence, including *Mb2515c* and flanking regions from MBO AF2122/97, and performing a blastn search against MBO Ravenel SRA reads (SRR13938830), which recapitulated the GenAPI findings showing no reads aligning over the *Mb2515c* gene and some flanking regions and suggests a deletion may have occurred.

## 4. Discussion 

Experimental and clinical studies referred to earlier show that MBO Ravenel is attenuated and elicits a robust immune response in cattle. Several genome-wide SNP studies in *M. tuberculosis* have shown to have sufficient resolution to develop trait-allele interactions. In the present study, we hypothesized that drivers of attenuation are enciphered in the genome of MBO Ravenel [11] and performed comparative genomic analysis of Ravenel against three clinical MBO strains—95-1315, 10-7428, and AF2122/97. Disease phenotype was assessed by experimental infections with either Ravenel or 95-1315. 

The present study utilized genomes of these select clinical strains to draw comparisons against the attenuated strain MBO Ravenel. This led to the identification of 32 highest confidence MBO Ravenel-specific missense SNPs, visualized by affected locus and position across the genome in Figure 5. Among these, a subset of 9 SNPs was selected based on functional annotation of impacted loci. These SNPs may contribute to reduced virulence and an attenuated phenotype as they affect cell wall synthesis- and transport-associated genes, pathways critical for the metabolism and intracellular survival of the pathogen.

### 4.1. Ravenel Elicits Robust Cell-Mediated Immune Response but Is Attenuated 

Experimental calf infection with Ravenel or 95-1315 revealed that the former strain did not produce granulomata but was isolated from lymph nodes. The animals used in this study were older than the ones referenced earlier. However, the low virulence of this strain in cattle was still surprising, given the high virulence of *M. bovis* Ravenel in mice, rabbits, and guinea pigs [7,13,14]. Furthermore, comparative immunopathogenesis revealed significant IFN-γ responses to rESAT-6/CFP10 or *M. bovis* PPD were elicited by *M. bovis* infection, regardless of the strain used. These findings are consistent with the observations of Khare et al. [11], who demonstrated similar attenuation of Ravenel in a vaccination challenge trial. Taken together, our findings confirm that Ravenel is attenuated in the bovine host deserving a deeper explanation for the observed phenotype.

In contrast to the present study, Khare et al. observed microscopic granulomas in tonsils from 7 of 10 Ravenel-infected cattle. This may be due to the intranasal route of inoculation used rather than the aerosol route of inoculation used in our study. Aerosolization of two different strains of *M. bovis*, using doses similar to those presented here, also failed to induce lesions or result in the colonization of tonsils [16]. In the present study, microscopic granulomas were not observed in the lungs of Ravenel-infected cattle, in contrast to those described by Khare et al. This may also be due to a difference in the route of inoculation, or differences in the number of passages of inoculum strains. 

### 4.2. Ravenel Carries SNPs in Genes Encoding Cell Wall Integrity 

The mycobacterial membrane protein [large] (*mmpL*) genes encode a broad family of transmembrane-transport proteins believed to be involved in fatty acid transportation by performing the function of flippases [32,33,34,35]. These housekeeping genes are essential for survival. Members of this family, such as MmpL3, have thus been proposed as a druggable target [36], and research into small molecule inhibitors has yielded mycobactericidal compounds [37]. Williams et al. reported a list of mutations in the mmpL3-mutant strains, of which none were identifiable in MBO Ravenel [37]. Instead, MBO Ravenel had missense mutation G2662A (V888I) in *mmpL8*. Unlike *mmpL3*′s role in mycobacteria survival, evidence is limited for *mmpL8* specifically. Nonetheless, being from the same family of proteins mutation in *mmpL8* might affect the transportation of pathogenesis-associated compounds.

Members of the MTBC possess unique Type VII secretion systems (ESX systems). These secretion systems contribute to virulence (ESX-1, -3, -5), nutrient uptake (ESX-5), metal homeostasis (ESX-3), and the export of PE/PPE family proteins (ESX-5) [33]. Disruption of these systems is associated with attenuation [34]. In MBO Ravenel’s ESX-5 system, we identified the polymorphism C1530T (T507M) in *eccC_5_*. The ATPase encoded by *eccC_5_* has three nucleotide-binding domains hypothesized to be essential for substrate recognition [35]. Ates et al. demonstrated that NBD mutations impaired bacterial growth [36]. The mutation in MBO Ravenel’s *eccC_5_* (T507M) falls directly adjacent to NBD-1 (K506), which may destabilize the binding and function of this virulence-associated system [36]. MBO Ravenel also has mutation G307A (A103T) in *espH*, a gene associated with the ESX-1 virulence system [37]. 

The biosynthesis of arabinogalactan by *aftB* is fundamental for the mycobacterial cell wall [38,39]. Raad et al. developed a *Corynebacterium glutamicum* Δ*aftB* mutant that caused outer membrane destabilization [39]. Jankute et al. demonstrated similar results in *M. smegmatis* [38]. Ravenel carries a mutation in *aftB* C1450T (H484Y), and whether this mutation results in deficient biosynthesis of arabinogalactan remains to be explored. 

Encoded by *rpfB*, resuscitation promoting factor (RPF) [40,41,42,43,44], cleaves peptidoglycan, employing E292 in its catalytic pocket [45,46]. MBO Ravenel carries the *rpfB* polymorphism A788G (E263G) that, while outside Squeglia et al.’s observed catalytic region [46], may conformationally affect the downstream pocket by replacement of a charged amino acid with a flexible glycine residue.

### 4.3. Ravenel Carries SNPs in Genes Associated with Respiration or Lipid Metabolism

Lipids are the primary carbon source for mycobacteria [47,48,49]. The *fadD* and *fadE* families of genes are involved in long-chain fatty acid synthesis in mycobacteria, encoding ligase, synthetase, and dehydrogenase. The enzyme encoded by *fadD29* converts long-chain fatty acids to the acyl adenylates required for phenol glycolipid (PGL) production, which in turn is required for the synthesis of the outer membrane of MTB [50]. In the present study, MBO Ravenel carried a mutation in *fadD29* A692G (N231S). PGL synthesis is required for mycobacterial viability, so missense changes warrant scrutiny [50,51]. MTB relies on host cholesterol import for carbon supply and survival during chronic infection through *fadE29.* Knockout studies by Thomas et al., and Gilbert et al., determined that *fadE28* and *fadE29* are essential for degrading cholesterol metabolites [52,53]. MBO Ravenel *fadE29* has missense mutation T1079G (V360G). The downstream effects of missense mutations in both *fadE29* and *fadD29* may contribute to alterations in bacterial viability. 

Polyketide synthase 5, encoded by *pks5*, has been identified as a virulence-associated biomarker of MTBC infection in cattle [54]. The product of *pks5* is thought to be involved in multimethyl-branched fatty acid synthesis required for lipooligosaccharide (LOS) biosynthesis [55]. Loss of *pks5* leads to severe MTB growth defects in animal models [56]. In MBO Ravenel, we observed a G1363A mutation (G455S) in *pks5*, though any effects on function require experimental testing.

The serine protease mycosin (MycP_1_) is a conserved membrane component of ESX-1 and ESX-5 systems [57]. Ohol et al. (2010) found that inhibiting MycP_1_ protease activity leads to increased ESX-1 substrate secretion [58]. C374T (T125I) in *mycP_1_* is, therefore, another SNP of interest in MBO Ravenel, as the destabilization of this protease is known to lead to dysregulation of the tightly controlled ESX-1 machinery. Considering that ESX-1-containing RD-1 is believed to be a major contributor to the substantial attenuation of MBO BCG [15,58,59,60], any disruption of this system by alternate means could explain virulence deficiencies despite RD-1’s presence.

Out of 32 genes with functional annotations containing missense mutations unique to MBO Ravenel, nine involved in cell wall integrity and critical metabolism have been described here. A single mutation could not reasonably explain the experimental attenuation observed in MBO Ravenel compared to fully virulent strains. However, the cumulative effect of many SNPs across the genome may have contributed to the impairment of pathogenesis. Only a subset of the observed SNPs has been discussed here, and with our stringent cutoffs and filtering, some informative changes may not be included in our analysis. The remaining 23 MBO Ravenel-specific missense mutations, as well as those in loci without annotated function or those in intergenic regions, likely also play some role. While not discussed here, insertions and deletions can have profound effects on gene function, and a table of 57 called indels is provided (Supplemental Table S1). Additionally, a GenAPI comparison of MBO Af2122/97 and MBO Ravenel suggested a genomic deletion and complete loss of Mb2515c, a predicted LuxR transcriptional regulator in MTB thought to contribute to pathogenesis through unknown mechanisms [61]. 

## 5. Conclusions

We aimed to compare the in vivo virulence of MBO Ravenel to MBO 95-1315 in the original bovine host and discover the pathways impacted by mutations across the MBO Ravenel genome. Cattle infection experiments supported previously identified differences in MBO Ravenel pathology in the bovine host. Detection of robust immune responses across animals, despite the absence of gross lesions, also supports the Ravenel strain’s deficiency in pathogenesis, causing only subclinical infection in contrast to the observed virulence from MBO 95-1315. The stringent set of identified highest-confidence missense polymorphisms in key genes involved with survival and pathogenesis of MBO provides a realistic genetic basis for attenuation. Which of these changes lead to functional impacts, if any, still requires elucidation. In summary, we confirm MBO Ravenel is attenuated and presents subclinically in the bovine host despite its broad genomic structural similarities to fully virulent MBO strains. A constellation of polymorphisms in key genes may instead explain its striking differences in disease phenotype when compared to virulent MBO 95-1315. 

## Figures and Tables

**Table 1:** Cattle Infection Study, Gross Pathology Results.

Treatment Group	Mean Disease Score ^a^		
	Tracheobronchial Lymph Nodes	Mediastinal Lymph Nodes	Lung
**Non-infected**	**0 ± 0** (0/5)	**0 ± 0** (0/5)	**0 ± 0** (0/5)
**MBO strain Ravenel infected**	**0 ± 0** (0/5)	**0 ± 0** (0/5)	**0.04 ± 0.04** (1/5)
**MBO strain 95-1315 infected**	1.4 ± 0.2 * (5/5)	1.4 ± 0.2 * (5/5)	1.2 ± 0.3 * (5/5)

^a^ At necropsy, tracheobronchial lymph nodes, mediastinal lymph nodes, and lungs were evaluated for lesions based on a scoring system adapted from Vordermeier et al., 2002. Mean disease scores are presented as mean ± sem. In addition, the number of animals with lesions/# of animals per group is provided in parentheses under disease scores. * Differs (*p* < 0.05) from other treatment groups relative to tissue.

**Table 2:** Cattle Infection Study, Recovery of *M. bovis* from lesions.

Treatment Group	Quantitative Culture (CFU/g) ^a^	Qualitative Culture ^b^		
		Tracheobronchial Lymph Node	Mediastinal Lymph Node	Lung
**Non-infected**	0 ± 0	0/5	0/5	0/5
**MBO Ravenel infected**	427 ± 1246	4/5	1/5	0/5
**MBO 95-1315 infected**	28,141 ± 9063 *	5/5	4/5	3/5

^a^ To eliminate bias based on the organ sampling site, entire tracheobronchial lymph nodes (other than a small ~1 g section for histology and qualitative culture) were homogenized and cultured for *M. bovis*. Values represent the mean (± standard error) CFU/g of tissue. * Differs from non- and *M. bovis* Ravenel-infected groups, *p* < 0.05. ^b^ For qualitative culture, values represent the number of tissues in which *M. bovis* was isolated for the number of animals per group.

**Table 3:** Nine MBO Ravenel-specific single nucleotide polymorphisms (SNPs) hypothesized to contribute to the attenuation of MBO Ravenel. High-confidence missense SNPs were extracted from MBO strain Ravenel (strain AF2122/97 as reference) and compared to strains 10-7428 and 95-1315.

Position	Gene Name	Gene Identifier	Mycobrowser Classification	Functional Annotation	DNA Change (Protein Change)
4305161	*mycP_1_*	*MB3913C*	Intermediary metabolism and respiration	membrane-anchored mycosin mycp1 (serine protease) (subtilisin-like protease) (subtilase-like) (mycosin-1)	C374T (T125I)
4283808	*espH*	*MB3897*	Cell wall and cell processes	esx-1 secretion-associated protein	G307A (A103T)
4229553	*mmpL8*	*MB3853C*	Cell wall and cell processes	conserved integral membrane transport protein	G2662A (V888I)
4208072	*aftB*	*MB3835C*	Cell wall and cell processes	possible arabinofuranosyltransferase	C1450T (H484Y)
2015643	*eccC_5_*	*MB1812*	Cell wall and cell processes	esx conserved component eccc5. esx-5 type vii secretion system protein	C1520T (T507M)
1129357	*rpfB*	*MB1036*	Cell wall and cell processes	Probable resuscitation-promoting factor	A788G (E263G)
1715469	*pks5*	*MB1554C*	Lipid metabolism	Probable polyketide synthase	G1363A (G455S)
3262864	*fadD29*	*MB2974C*	Lipid metabolism	fatty-acid-amp ligase fadd29 (fatty-acid-amp synthetase) (fatty-acid-amp synthase)	A692G (N231S)
3929690	*fadE29*	*MB3573C*	Lipid metabolism	Probable Acyl-CoA dehydrogenase	T1079G (V360G)

## Data Availability

All genomic data are available on NCBI under accession numbers provided in the manuscript.

## Supplementary Materials

The following supporting information can be downloaded at: https://www.mdpi.com/article/10.3390/pathogens11111330/s1, Table S1: Ravenel(Rav)-Unique(Uni)-Missense(Mis)-Putative Function Associated(Fun)-Present In Correctly Assembled Region Of Genome(Cor)-32SNPs; Table S2: Ravenel(Rav)-Unique(Uni)-Missense(Mis)-Putative Function Associated(Fun)-Present In Correctly Assembled Region Of Genome(Cor)-Identifiable In Annotated Genome InP ATRIC(PAT)-9SNPs; Table S3: Ravenel Gaps Between Contigs
